# Assessment of the Entomopathogenic Potential of Fungal and Bacterial Isolates from Fall Armyworm Cadavers Against *Spodoptera frugiperda* Caterpillars and the Adult Boll Weevil, *Anthonomus grandis*

**DOI:** 10.1007/s13744-024-01159-0

**Published:** 2024-05-07

**Authors:** Lidiane Maria Dos Santos Moreira, Luciene Silva Marinho, Robério Carlos Santos Neves, Ricardo Harakava, Layara Alexandre Bessa, Luciana Cristina Vitorino

**Affiliations:** 1 Instituto Goiano de Agricultura (IGA), Montividiu, GO Brazil; 2 Instituto Biológico de São Paulo, São Paulo, SP Brazil; 3 Lab of Biodiversity Metabolism and Genetics, Instituto Federal Goiano, Rio Verde Campus, Rio Verde, GO Brazil; 4 Simple Agro Corporation, Rio Verde, GO Brazil; 5 Lab of Agricultural Microbiology, Instituto Federal Goiano, Rio Verde Campus, Rio Verde, GO Brazil

**Keywords:** Biological inputs, Coleoptera, Lepidoptera, Pest biocontrol microorganisms, Sustainable agriculture

## Abstract

Increased attention is being focused on the biological control of agricultural pests using microorganisms, owing to their potential as a viable substitute for chemical control methods. Insect cadavers constitute a potential source of entomopathogenic microorganisms. We tested whether bacteria and fungi isolated from *Spodoptera frugiperda* (JE Smith) cadavers could affect its survival, development, egg-laying pattern, and hatchability, as well as induce mortality in *Anthonomus grandis* Boheman adults. We isolated the bacteria *Enterobacter hormaechei* and *Serratia marcescens* and the fungi *Scopulariopsis* sp. and *Aspergillus nomiae* from fall armyworm cadavers and the pest insects were subjected to an artificial diet enriched with bacteria cells or fungal spores to be tested, in the case of *S. frugiperda*, and only fungal spores in the case of *A. grandis*. *Enterobacter hormaechei* and *A. nomiae* were pathogenic to *S. frugiperda*, affecting the survival of adults and pupae. The fungus *Scopulariopsis* sp. does not affect the survival of *S. frugiperda* caterpillars and pupae; however, due to late action, moths and eggs may be affected. *Aspergillus nomiae* also increased mortality of *A. grandis* adults, as well as the development of *S. frugiperda* in the early stages of exposure to the diet, as indicated by the vertical spore transfer to offspring and low hatchability. *Enterobacter hormaechei* and *A. nomiae* are potential biocontrol agents for these pests, and warrant further investigation from a toxicological point of view and subsequently in field tests involving formulations that could improve agricultural sustainability practices.

## Introduction

Entomopathogenic microorganisms are an important option for naturally controlling major crop pests (Ruiu 2015; Ortiz-Urquiza et al. 2015). Currently, biocontrol agents play a significant role in agriculture as an environmentally friendly response to control the growth of plant pathogens, in line with the increased demands for sustainable agriculture (Elnahal et al. 2022; Tariq et al. 2020; Tracy  2015). Thus, biopesticides have great potential for application not only in organic agricultural practices, but also in large-scale conventional practices (Ramakuwela et al. 2020; Jaber and Enkerli 2016; Gardener and Fravel 2002). As a result, the market for microbial inoculants has grown worldwide at an annual rate of approximately 10%, not only due to their effectiveness but also because microbial inoculants are considered much safer than chemical pesticides (Berg 2009).

Thus, this market requires an increase in the availability of microbial biopesticides for commercial use (Bonaterra et al. 2022). This has a direct impact on research on microbial strains and metabolites for use in the composition of inoculant formulations (e.g., Sabbahi et al. (2022); Haris et al. (2021); Volpiano et al. (2018); Polonio et al. (2015)). Biocontrol microorganisms have already been prospected from several environmental samples, as well as rhizospheric or endophytic microorganisms from native, medicinal, or commercial plants (Giannelli et al. 2022; Da Silva et al. 2021, 2018; Hussain et al. 2016). Alternative sources such as insect cadavers have been suggested as a source for prospecting entomopathogenic strains (Abdel-Raheem 2022; Sharma et al. 2020). Bearing this in mind, we tested the hypothesis that bacteria and fungi isolated from cadavers of *Spodoptera frugiperda* (JE Smith) can behave as generalist entomopathogens, affecting the survival and fitness of *S. frugiperda*. We also tested whether the fungal isolates could cause infection in other insect pests of a different order, such as *Anthonomus grandis* Boheman.

Species of the *Spodoptera* complex can cause considerable yield losses owing to their defoliation ability (De Groote et al. 2020). *Spodoptera frugiperda*, the fall armyworm, is a polyphagous pest with no diapause and high migration capacity, in addition to a broad host range, voracious larval feeding, and high fecundity (Wang et al. 2020a, b; Wang et al. 2020a, b). Agricultural losses from *S. frugiperda* affect many countries, as it is now considered an invasive pest in Africa, Asia, and Oceania (Ayra-Pardo et al. 2021). The caterpillar of this species is the main pest of maize crops, attacking mainly young plants that may not even produce cobs, causing commercial losses of 15–37% (Cruz 1993; Mitchell 1979; Ferreira Filho et al. 2010). Currently, *S. frugiperda* is managed mainly with broad-spectrum chemical insecticides, which are harmful to beneficial arthropods (Burtet et al. 2017; Guillebeau and All 1991). Although insecticidal control remains a primary tactic to manage *S. frugiperda*, its extensive use has increased the resistance of this pest to conventional insecticidal compounds. In recent decades, *Bacillus thuringiensis* toxins have emerged as the main alternative to chemical pesticides for the management of this pest control, but over time, this species has also developed resistance to Cry proteins (Gutiérrez-Moreno et al. 2019; Farias et al. 2014). In fact, the increased use of plants with this technology may have increased the number of outbreaks of this invasive species in crops around the world (Montezano et al. 2018), which makes the prospecting of new biocontrol agents to manage this insect more urgent. From this perspective, the use of nucleopolyhedroviruses has recently emerged as an important tool to control *Spodoptera* populations and reduce resistance issues (Sajid et al. 2021).

The same is true for pest insects of the order Coleoptera. The cotton boll weevil (*A. grandis*), for example, is one of the most well-known and historically important insect pests in the Americas. Starting in the 1970s, a major research effort, implemented through the National Boll Weevil Eradication Program, was necessary to eradicate the boll weevil from the southeastern USA, but current studies indicate potential for increasing the geographic distribution of this pest and modification of its global habitats with the advent of climate change (Jin et al. 2022). It is currently considered the main cotton crop pest in Brazil (Oliveira et al. 2013). This species feeds on buds and flowers of cotton crops, preventing normal cotton boll opening, and destroying its fibers and seeds. Damage caused ​by this pest in the absence of control measures can reach 58–84% of production (Ramalho and Santos 1994). Economic losses, even with the use of chemical control, can range between US$ 51 and 74 million per year in Brazil (Oliveira et al. 2013). The cotton boll weevil has become resistant to a broad range of chemicals (Perkin et al. 2023; Oliveira-Marra et al. 2019), which incentivizes the prospection of ecological alternatives to control this pest.

*Metarhizium anisopliae* and *Beauveria bassiana* in combination with deltamethrin were already suggested for control of *A. grandis* (Bleicher et al. 1994; Nussenbaum and Lecuona 2012). *Bacillus thuringiensis*, *Metarhizium rileyi*, *M. anisopliae*, and *B. bassiana* successfully control *S. frugiperda* in tests conducted in vitro (Abbas et al. 2022; Idrees et al. 2022; Montecalvo and Navasero 2021; Guo et al. 2020). Due to the limited number of products available for biocontrol of these pests, we evaluated the pathogenic characteristics of microorganisms obtained from cadavers of *S. frugiperda* administered through an enriched artificial diet. We analyzed their effects on the mortality of caterpillars, pupae, and adults, on development in the early stages of exposure, and on egg laying and hatchability. We also fed adult *A. grandis* insects with an enriched artificial diet to evaluate the mortality induced by the tested microorganisms.

## Materials and Methods

### Isolation and Purification of Potentially Entomopathogenic Microorganisms

*Spodoptera frugiperda* caterpillar cadavers were sequentially obtained in five weekly collections from the entomological crop collection of the Laboratory of Agricultural Entomology of the Goiano Institute of Agriculture (IGA), where insect cadavers were preserved under aseptic conditions. At each collection, three insect cadavers were randomly sampled and superficially disinfected to remove epiphytic microorganisms. Dead caterpillars were immersed in sodium hypochlorite (2%) for 1 min, in 70% alcohol for 30 s, and rinsed five times in sterilized distilled water. The sterilization efficiency was tested by inoculating 100 μL of last rinsing water on nutrient agar (NA) (meat extract, 1 g; yeast extract, 2 g; peptone, 5 g; sodium chloride, 5 g; agar, 15 g; final pH = 6.8 ± 0.2), in triplicate. In a laminar flow cabinet, the sampled insects had their abdomens aseptically sectioned using a scalpel, and the fragments were spread on the surface of Petri dishes containing tryptic soy agar (TSA) culture medium (tryptone, 15 g; soy papain digest, 5 g; sodium chloride, 5 g; agar, 15 g; final pH = 7.3 ± 0.2). The plates were incubated for 7 days at 25 ± 2 °C.

The growth of microbial colonies from the fragments was monitored daily and, when present, the colonies were purified on TSA. Bacterial colonies were purified by depletion streaking, and fungi by plating young mycelium, with four microbial isolates, two bacterial and two fungal, being obtained.

### Molecular Identification of Fungal Isolates

Bacterial isolates were grown in nutrient broth for 48 h for prior molecular identification, and fungal isolates in potato dextrose (PD) broth (dextrose, 20 g; potato immersion broth, 400 mL, in 1 L of distilled water, final pH = 6.6 ± 0.2) for 7 days. Subsequently, genomic DNA was extracted in triplicate from each isolate, according to the methodology proposed by Cheng and Jiang (2006), using a Miniprep extraction kit (Axygen Biosciences, Union City, CA, USA). Identification was performed after amplification and purification by sequencing the 16S region for bacteria and the 28S region for fungi. Sequencing was performed by the Sanger method, and for phylogenetic inference, sequences were paired by similarity with sequences from the GenBank, via nucleotide Basic Local Alignment Search Tool (BLASTn) (Altschul et al. 1990), using homology > 98%. Subsequently, the bacterial sequences were concatenated and aligned to homologous sequences of six bacterial species (*Enterobacter hormaechei*, *Enterobacter cloacae*, *Enterobacter carcinogenus*, *Serratia marcescens*, *Serratia nematodiphila*, and *Serratia ureilytica*) extracted from GenBank. The fungal sequences were concatenated and aligned to sequences of 11 fungal species (*Aspergillus nomiae*, *Aspergillus flavus*, *Aspergillus novoparasiticus*, *Aspergillus carbonarius*, *Aspergillus niger*, *Scopulariopsis alboflavescens*, *Scopulariopsis flava*, *Scopulariopsis brevicaulis*, *Scopulariopsis koningii*, and *Scopulariopsis candida*) also obtained from the GenBank. The sequences were aligned using Clustal Omega (Sievers and Higgins 2014).

The evolutionary model of the sequences was selected using the Bayesian Information Criterion (BIC) in jModelTest 2 (Darriba et al. 2012). The selected model was TrN + G, with gamma shape of 0.4450 for bacteria and TrN for fungi. Phylogenetic trees were independently inferred for bacteria and fungi using methods based on Bayesian inference in MrBayes v.3.2.6. (Ronquist et al. 2012). Each tree underwent four independent runs, with 10 × 10^6^ generations assigned to the chains and a posteriori probability distribution every 500 generations. The first 2500 trees sampled were discarded before calculating the consensus trees, one for bacteria and one for fungi, to ensure chain convergence. The phylogenetic trees with Bayesian maximum likelihood were visualized and edited in FigTree v 1.4.4 (Rambaut 2014). Sequences of the species *Lactobacillus lactis* and *Penicillium chrysogenum* were used as outgroups, in the inferred trees for bacteria and fungi, respectively.

### Obtaining and Rearing of *S. frugiperda* and *A. grandis*

This study did not directly use plant materials, nor did it use caterpillars or adult insects collected from natural populations; however, all necessary licenses and permissions were obtained for the development of the study, including a license to collect (SISBio—769521) and permission for in vitro cultivation (ordinance CMRV-DGPI-04/2023 of the IFGoiano campus Rio Verde). Thus, *S. frugiperda* larvae required for pathogenicity testing were collected from the rearing facility of the IGA Entomology Laboratory. First-instar larvae were kept in 50-mL plastic pots (one caterpillar per pot) with perforated lids from egg hatching until the experiment was set, being fed an artificial diet (beans, 56.25 g; wheat germ, 45 g; brewer’s yeast, 28.15 g; soy protein, 22.50 g; casein, 22.50 g; agar, 17.50; sorbic acid, 1.35 g; ascorbic acid, 2.70 g; methylparaben, 2.25 g; and tetracycline, 0.09 g), prepared according to Cruz (2000). Adult *A. grandis* individuals were manually collected from cotton plantations installed in the IGA commercial plantation area (17°26′43.51″S, 51°08′47.55″W) for testing. These insects were kept in ventilated cages and, for the experimental set-up, transferred to 50-mL plastic pots containing the artificial diet, same as *S. frugiperda*.

### Pathogenicity Tests Against *S. frugiperda* and *A. grandis*

These tests were individually conducted using bacterial cultures in liquid medium and fungal spore suspensions. We tested the effect of bacterial and fungal isolates on *S. frugiperda* and only the effect of fungal isolates on *A. grandis*. Bacterial cultures were prepared in Luria Bertani (LB) broth (tryptone,10 g; yeast extract, 5 g; sodium chloride, 5 g; final pH = 7.0 ± 0.2), incubated at 25 ± 2 °C, for 48 h. After this growth period, the cultures were plated on TSA to count colony-forming units (CFU) mL^−1^ after 24 h. The cultures were standardized to CFU and used at a concentration of 10^7^ CFU mL^−1^.

A fungal spore suspension was prepared by growing young mycelia of the isolates on plates containing potato dextrose agar (PDA) and incubated at 25 ± 2 °C until abundant sporulation occurred. The cultures were then flooded with saline solution (8.5 g) containing Tween 80 (1 mL), and the spores were scraped off with a Drigalski spatula. Spore concentration was evaluated by spore counting in a Neubauer chamber, and was standardized to 10^7^ mL^−1^.

The effect of each microbial culture was assessed in isolation. For this, 50 µL of each bacterial culture or spore solution was pipetted directly onto the artificial diet (30 mL) as described above for first-instar *S. frugiperda* larvae or adult *A. grandis* insects. For technical reasons, we only adopted fungal treatment against the boll weevil. The plastic jars containing the insects were kept under controlled temperature (26 ± 2 °C), relative humidity (70 ± 10%), and photoperiod (LD 12:12). Insects were monitored every 2 days until completion of the larval and pupal stages of *S. frugiperda* development. After 3 days of development, the pupae were removed from the plastic jars and placed in cages (26 ± 2 °C, separated by treatment) and monitored every 2 days until completing the pupal stage, the adult stage, and producing *S. frugiperda* offspring. The offspring were removed from the cages and transferred to plastic pots containing the artificial diet (30 mL) without inoculum, where they remained until hatching. Mortality data and the stage of each dead experimental unit were assessed. Total monitoring time lasted 40 days after treatment (DAT) for *S. frugiperda* and 30 DAT for *A. grandis*. Mortality observed up to 15 days of exposure (1, 5, 10, and 15 DAT) was assessed separately from mortality at the end of the monitoring period. Mortality observed up to 15 days was considered mortality in the initial phase of exposure, but for *S. frugiperda*, the effects were monitored throughout development and *A. grandis* only in the adult phase. Larvae grown on a diet without inoculation were used as a control for the test with *S. frugiperda*. In the experiment with *A. grandis*, the control treatment consisted of adults that were not inoculated via diet.

Dead *S. frugiperda* larvae, pupae, or moths, and *A. grandis* adults were collected and counted. Subsequently, these cadavers were superficially decontaminated and distributed in Petri dishes containing PDA for re-isolation of the tested microorganisms.

### Experimental Design and Statistical Analysis

Experiments were conducted in isolation for each insect. The data on *S. frugiperda* were obtained in a completely randomized design with a 4 × 4 factorial scheme, i.e., four microbial treatments (two bacteria and two fungi) and 4 time points to evaluate their impact on the survival and development of caterpillars and pupae at the initial periods of exposure (1, 5, 10, and 15 DAT). Each treatment was evaluated in five replicates consisting of ten insects. The data were subjected to the Kolmogorov–Smirnov normality test and analysis of variance (ANOVA), with the *F* test at a 5% probability level. The *A. grandis* data were also analyzed by regression for DAT and *S. frugiperda* data were evaluated by Tukey’s test (*p* < 0.05) only at 10 and 15 DAT. The effect of the type of inoculum, when significant, was separately compared for bacteria and fungi by Tukey’s test (*p* < 0.05). Microorganisms were evaluated throughout the development cycle of *S. frugiperda* for the following variables: dead larvae count, dead pupae count, deformed pupae count, viable pupae count, dead moth count (during the first 5 days of this phase), total offspring count, and hatched offspring count.

Data on *A. grandis* were also obtained by completely randomized design, with two microbial inoculation treatments (2 fungi), evaluated only in the adult stage. Each treatment was evaluated in 13 replicates of ten insects. The data were analyzed only for the number of dead insects, and ANOVA and regression tests were used to evaluate the effect of treatments with microorganisms at the initial period of exposure (1, 5, 10, and 15 DAT). In the case of the effect of the type of inoculum (fungus species), the means were compared using Tukey’s test (*p* < 0.05). R Version 4.2.1 (R Core Team R 2021) was used for all analyses.

## Results

### Identification of Isolates

**Fig. 1:**
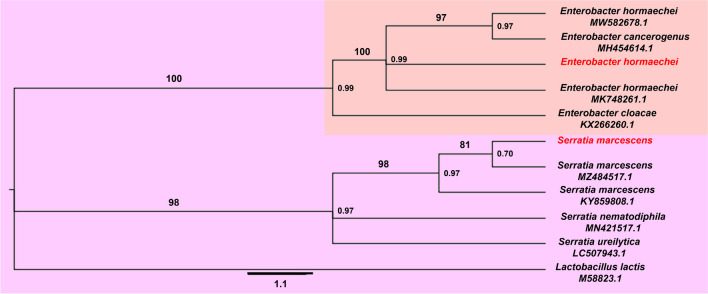
Similarity tree between different species of the genera *Enterobacter* and *Serratia* and two entomopathogenic bacterial strains isolated from *Spodoptera frugiperda* cadavers. Species in red indicate the isolates and their most likely classification. Phylogenic data were recovered based on the 16S region, with *Lactobacillus lactis* being used as outgroup. The numbers on the nodes show probability and the numbers above the branches show support. The scale bar below the tree represents the genetic distance

The phylogenic data recovered identified one of the bacterial strains as *E. hormaechei*, based on its similarity to the MK748261.1 and MW582678.1 strains. The other bacterial strain showed high similarity with MZ484517.1 and KY859808.1, from *S. marcescens*, constituting a distinct clade that is strongly supported and genetically distant from other *Serratia* species, such as *S. nematodiphila* and *S. ureilytica* (Fig. 1).

**Fig. 2:**
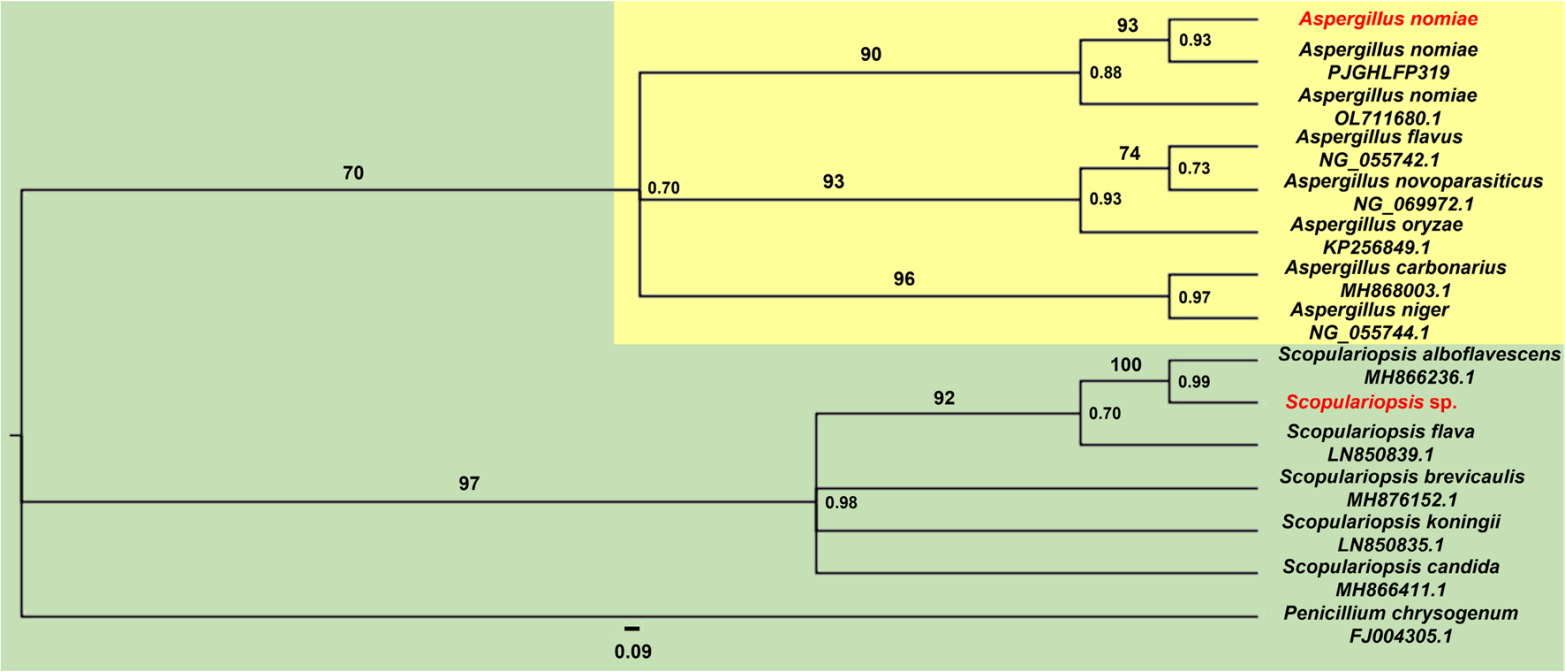
Similarity tree between different species of the genera *Aspergillus* and *Scopulariopsis* and two entomopathogenic fungal strains isolated from *Spodoptera frugiperda* cadavers. Species in red indicate the isolates and their most likely classification. Phylogenic data were recovered based on the 28S region, and *Penicillium chrysogenum* was used as outgroup. The numbers on the nodes show probability and the numbers above the branches show support. The scale bar below the tree represents the genetic distance

The phylogenic data recovered for fungal isolates identified the strains as *Scopulariopsis* sp. and *A. nomiae*. Thus, it was not possible to classify the species of *Scopulariopsis* because of its similarity not only to *S. alboflavescens*, but also to *S. flava*. In contrast, the *A. nomiae* clade is strongly supported by inference (Fig. 2).

### Pathogenicity Test Against *S. frugiperda* larvae and *A. grandis* Adults at the Initial Period of Exposure

**Fig. 3:**
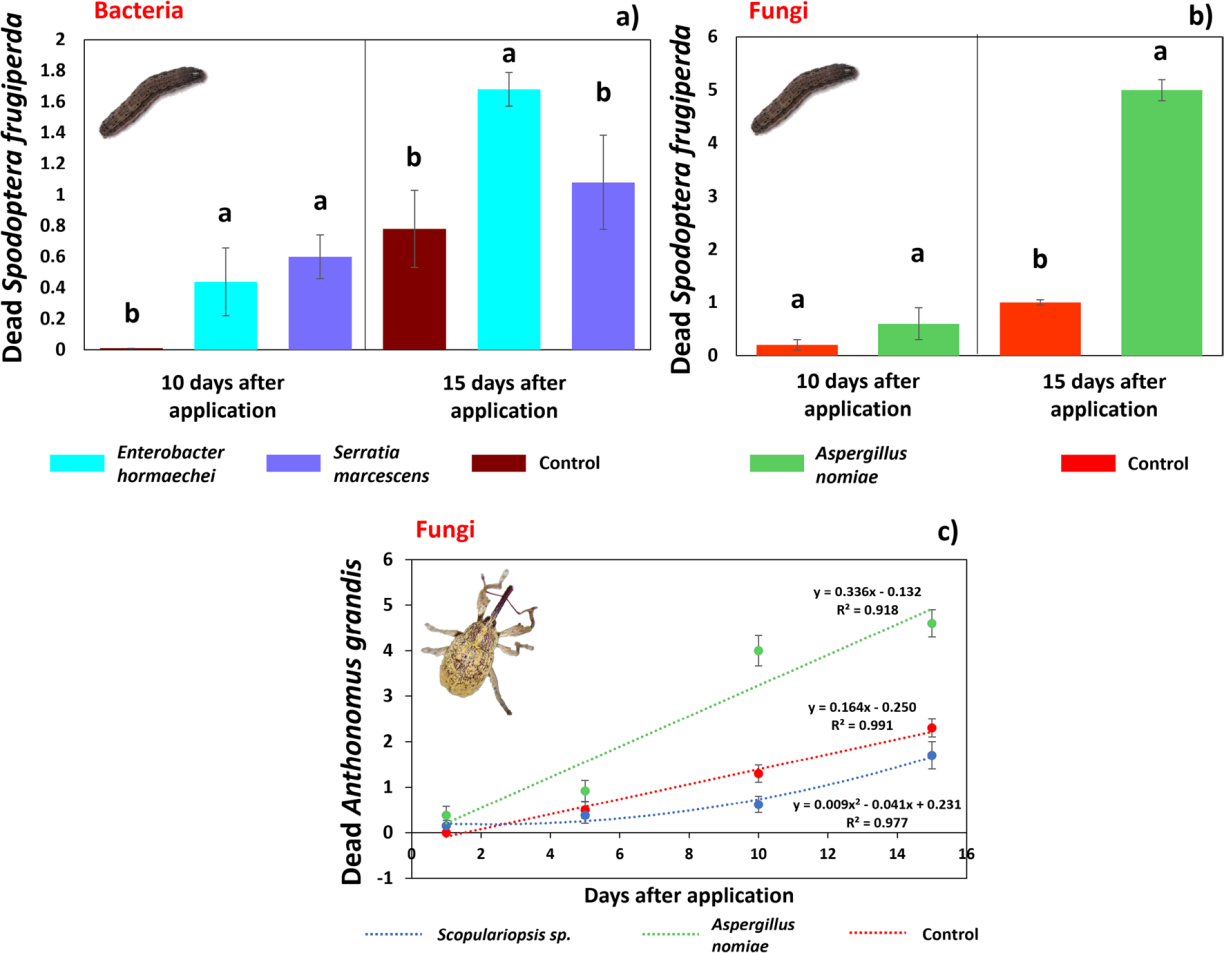
Mortality of *Spodoptera frugiperda* caterpillars and *Anthonomus grandis* adults subjected to a diet containing the bacteria *Enterobacter hormaechei* and *Serratia marcescens* and spores of the fungi *Scopulariopsis* sp. and *Aspergillus nomiae*. Effect of bacteria (a) and fungi (b) on the mortality of *S. frugiperda* in the initial phase of exposure (up to 15 days after treatment), and mortality of *A. grandis* adults treated with spores of the same fungi (c)

In the initial period of exposure, we did not observe mortality of *S. frugiperda* at 1 and 5 DAT; however, an effect was observed in larvae following bacterial treatments (*E. hormaechei* and *S. marcescens*), 10 days after exposure. During this period, no dead caterpillars were observed in the control treatment; however, no significant difference was observed in the virulence of the two bacteria evaluated against *S. frugiperda*. At 15 DAT, the treatments with *E. hormaechei* showed the highest mean mortality (1.68) (*F* = 10.29; D.F. = 14; *p* = 0.002) (Fig. 3a). In treatments with fungi, no immediate mortality effect was observed for *Scopulariopsis* sp. Caterpillars exposed to *A. nomiae* spores presented the pathogenicity effect at 15 DAT, so that the highest mean mortality was evidenced at 15 DAT (4.90) (*F* = 6.29; D.F. = 14; *p* = 0.013) (Fig. 3b). Similar behavior was verified when *A. grandis* adults were fed with spores of the tested fungi, i.e., only *A. nomiae* showed pathogenicity over the initial exposure time, with the highest mean mortality at 15 DAT (4.60) (Fig. 3c). Thus, larvae of *S. frugiperda* and adults of *A. grandis* appear to be insensitive to the action of *Scopulariopsis* sp. in their early exposure phase (first 15 days DAT), thereby indicating that they are relatively less influenced compared to the control treatment.

### Treatment Effect on Life Cycle of *S. frugiperda*

**Fig. 4:**
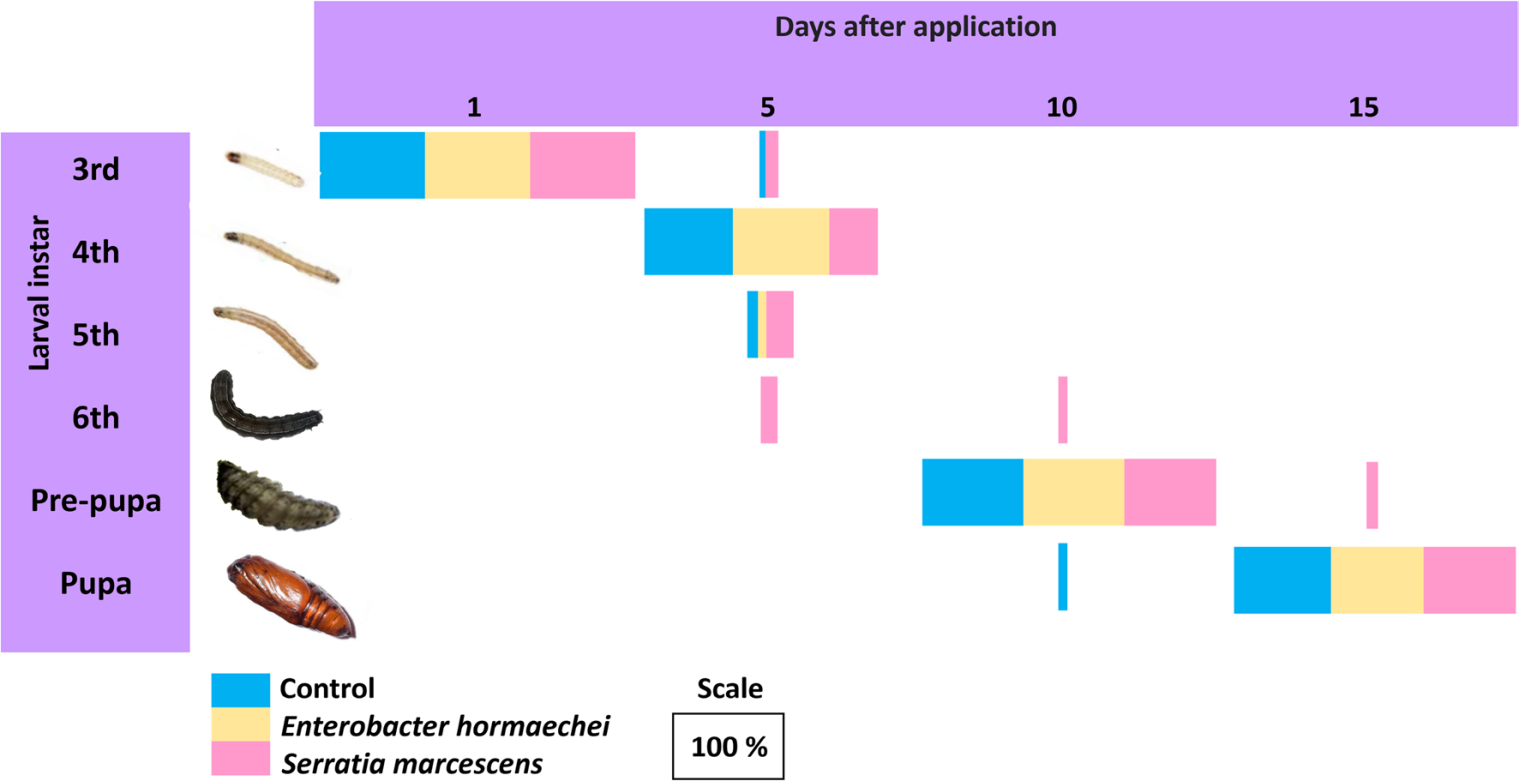
Percentage of caterpillars and pupae of *Spodoptera frugiperda* subjected to a diet containing the bacteria *Enterobacter hormaechei* and *Serratia marcescens* and distributed in different stages of development (3rd, 4th, 5th and 6th instar, pre-pupa and pupa), over four evaluation periods (1, 5, 10, and 15 DAT)

All *S. frugiperda* were third-instar at the first DAT. At 5 DAT, control caterpillars and caterpillars treated with *E. hormaechei* were mostly fourth-instar, whereas in the treatment with *S. marcescens*, a small percentage had already reached the sixth-instar stage (Fig. 4). At 10 DAT, an even smaller percentage of the caterpillars treated with *S. marcescens* were still sixth-instar, while those treated with *E. hormaechei* were 100% in the pre-pupal stage and the control ones had already started their transition to pupae. At 15 DAT, control caterpillars and those treated with *E. hormaechei* were 100% in the pupal stage, whereas 8% of those treated with *S. marcescens* were still in the pre-pupal stage. Therefore, *S. marcescens* appears to have stimulated the development of the caterpillars immediately after being included in the diet, but delayed this development throughout the exposure period.

**Fig. 5:**
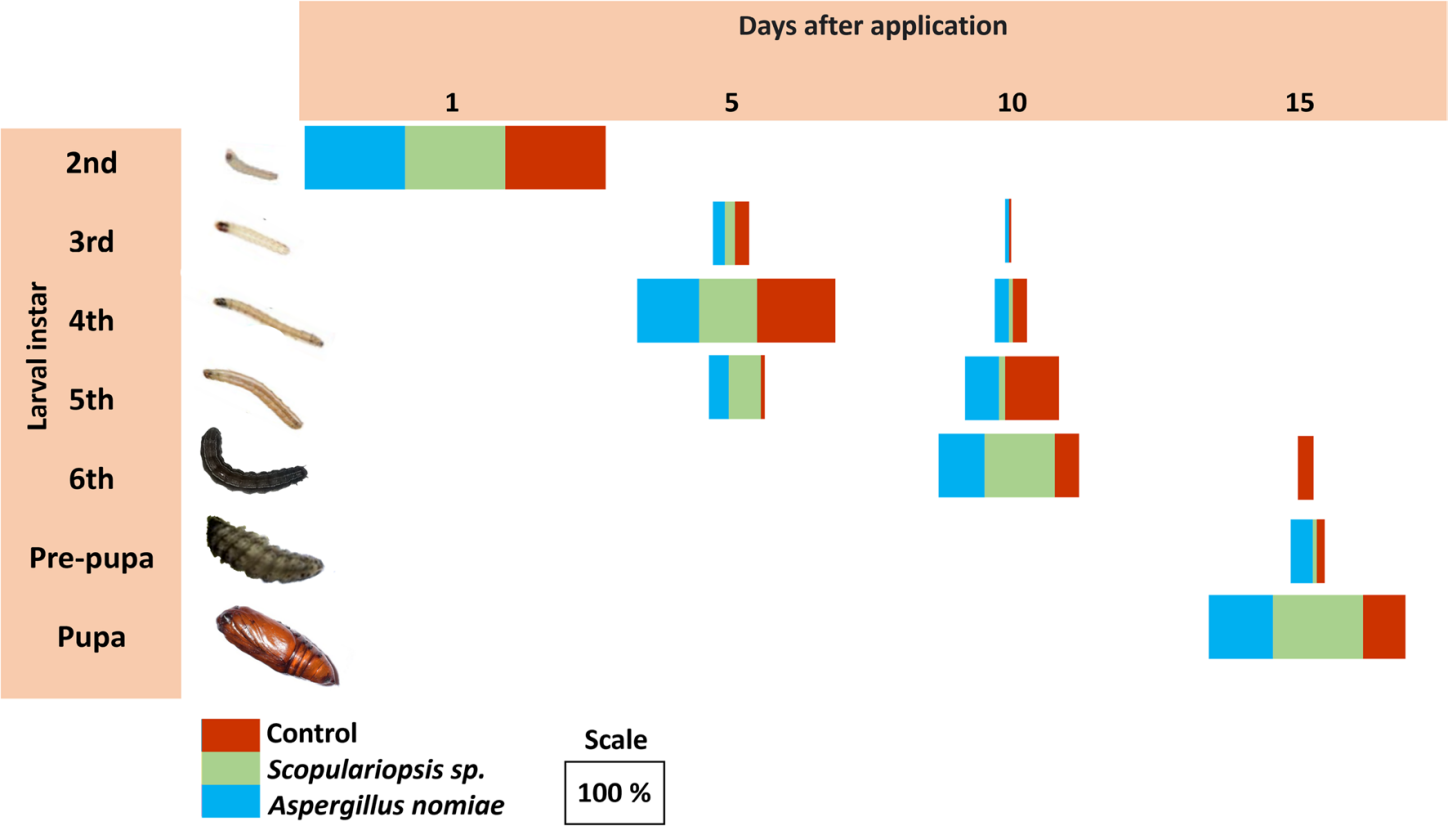
Percentage of caterpillars and pupae of *Spodoptera frugiperda* subjected to a diet containing spores of the fungi *Scopulariopsis* sp. and *Aspergillus nomiae* and distributed in different stages of development (2nd, 3rd, 4th, 5th, and 6th instar, pre-pupa, and pupa), over four evaluation periods (1, 5, 10, and 15 DAT)

At the first DAT with fungal treatments, all *S. frugiperda* were in second-instar (Fig. 5), and at 5 DAT, the highest percentage of the caterpillars from the three treatments were in fourth-instar. However, more caterpillars subjected to fungal treatments were in fifth-instar (27% for *Scopulariopsis* sp. and 23% for *A. nomiae*). At the 10 DAT, 60% of caterpillars in the control group were fifth-instar, whereas a higher percentage of caterpillars treated with spores of *Scopulariopsis* sp. and *A. nomiae* were already in sixth-instar (respectively 89 and 45%). However, at the last DAT, some caterpillars of the control group remained in sixth-instar (30%), whereas most of those treated with *A. nomiae* (70%) and *Scopulariopsis* sp. (95%) were already in the pupal stage. Thus, fungal spore treatments slightly accelerated the development of caterpillars, especially those of *Scopulariopsis* sp.

### Mortality at Different Stages of *S. frugiperda* Development

**Fig. 6:**
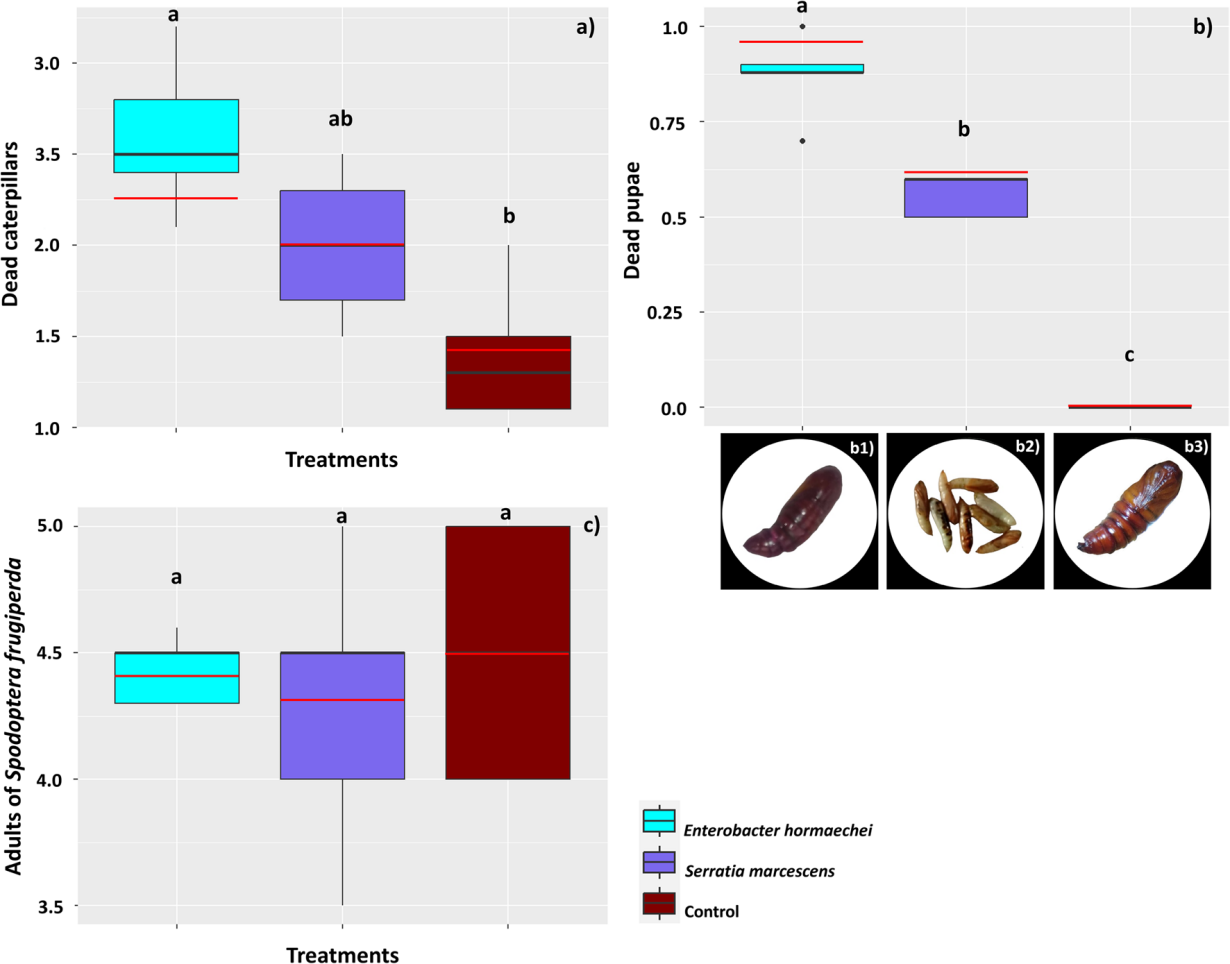
Mortality of *Spodoptera frugiperda* subjected to a diet containing the bacteria *Enterobacter hormaechei* and *Serratia marcescens* at different stages of development: larvae (**a**), pupae (**b**), and moths (**c**). The black horizontal bars within the boxplots represent the median number of dead insects obtained from five replicates with ten initial insects, and the red horizontal bars represent the average. The vertical bars are the maximum and minimum values and points outside the box are outliers. Above the boxes, equal letters represent statistically similar means (Tukey’s test, *p* < 0.05). In (b), the photos show dead pupae following treatment with *Enterobacter hormaechei* (b1) and *Serratia marcescens* (b2), as well as a healthy pupa (b3), verified in the control treatment

These results comprise observations carried out throughout the development of *S. frugiperda*, up to 40 DAT. The bacterial treatments affected the mortality of *S. frugiperda* (*F* = 11.13; D.F. = 14; *p* = 0.001), so that the highest mean mortalities were observed in caterpillars subjected to the diet with *E. hormaechei* (mean = 2.5 ± 0.41 dead caterpillars, equivalent to 25% mortality), followed by those treated with *S. marcescens* (mean = 2.0 ± 0.41 dead caterpillars, equivalent to 20% mortality) (Fig. 6a). The treatments also affected pupal mortality (*F* = 198.93; D.F. = 14; *p* = 0.000), with a higher mean number of dead pupae (mean = 0.88 ± 0.10 dead pupae, equivalent to 9% mortality) in the *E. hormaechei* treatment than in the *S. marcescens* treatment (mean = 0.60 ± 0.10 dead pupae, equivalent to 6% mortality) (Fig. 6b). However, *S. frugiperda* adults showed no differences between the mean number of dead moths in the treatments with the bacteria or the control (*F* = 0.27; D.F. = 14; *p* = 0.77) (means = 4.4 ± 0.13, 4.3 ± 0.57, and 4.5 ± 0.50 dead moths, respectively) (Fig. 6c).

**Fig. 7:**
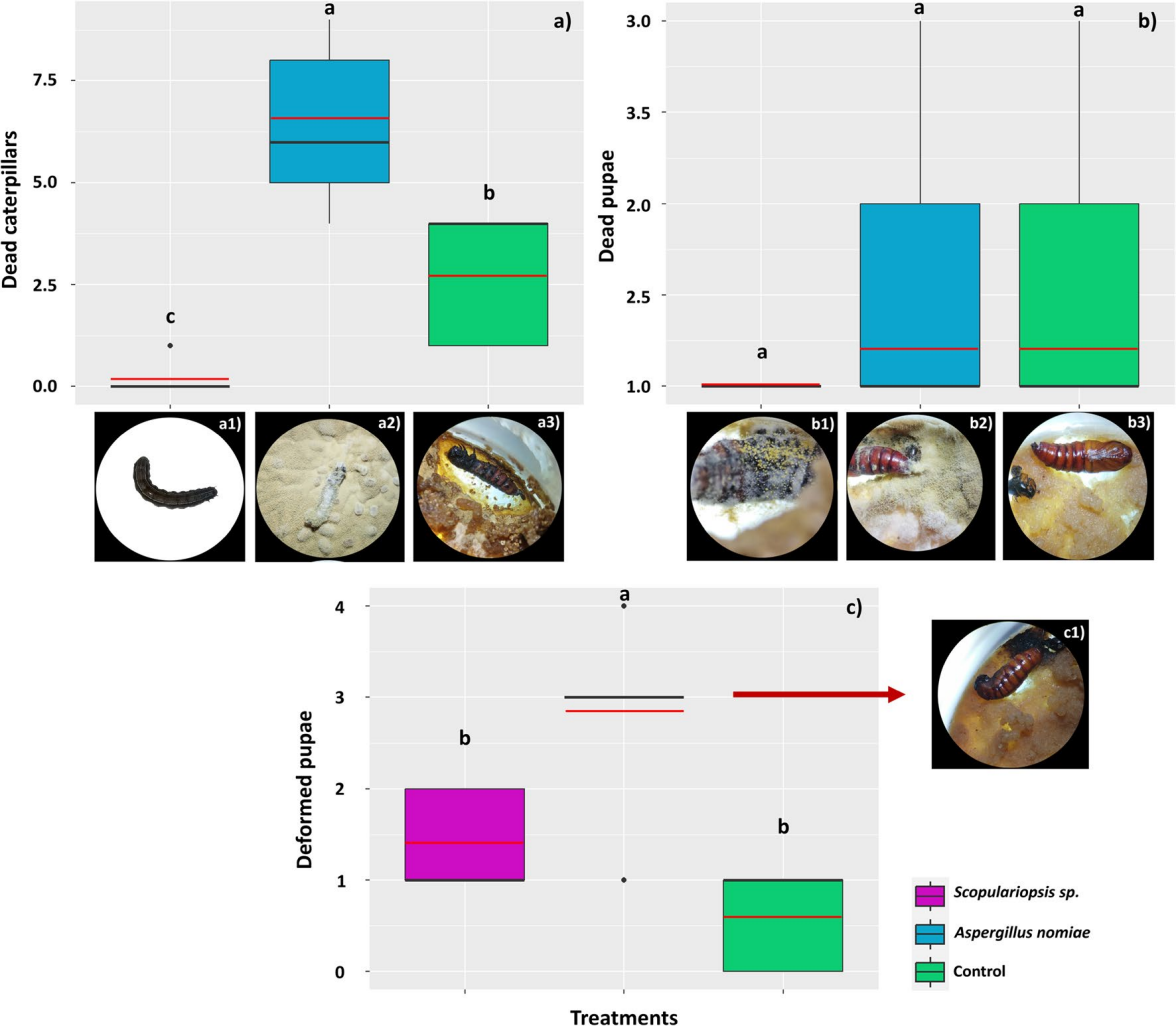
Mortality of *Spodoptera frugiperda* subjected to a diet containing spores of the fungi *Scopulariopsis* sp. and *Aspergillus nomiae* at different stages of development: larvae (**a**), pupae (**b**), and deformed pupae (**c**). The black horizontal bars within the boxplots represent the median number of dead insects obtained from five replicates with ten initial insects, and the red horizontal bars represent the average. The vertical bars are the maximum and minimum values and points outside the box are outliers. Above the boxes, equal letters represent statistically similar means (Tukey’s test, *p* < 0.05). In (**a**), the photos show dead caterpillars observed respectively in treatments with *Scopulariopsis* sp. (a1), *Aspergillus nomiae* (a2), and control (a3). In (**b**), the photos show dead pupae due to colonization with the tested fungi (b1 and b2), as well as a dead pupa observed in the control treatment (b3) and in (**c**), the photo shows the appearance of the deformed pupae observed in the treatment with *Aspergillus nomiae*

Fungal spore treatments affected the mortality of *S. frugiperda* throughout the different developmental stages. There was an expressive mean mortality of caterpillars (*F* = 20.19; D.F. = 14; *p* < 0.001) in the treatments with *A. nomiae* (mean = 6.4 ± 2.07 dead caterpillars, equivalent to 64% mortality), followed by the treatments with *Scopulariopsis* sp. (mean = 0.2 ± 0.40 dead caterpillars or 2% mortality) (Fig. 7a). Regarding dead pupae, there was no significant difference between fungal spore treatments (*F* = 1.12; D.F. = 14; *p* = 3.56) (Fig. 7b). Some deformed pupae were identified, so that *A. nomiae* significantly increased the frequency of pupal deformation (*F* = 10.33; D.F. = 14; *p* = 0.002) (mean = 2.8 ± 0.15 deformed pupae) (Fig. 7c).

**Fig. 8:**
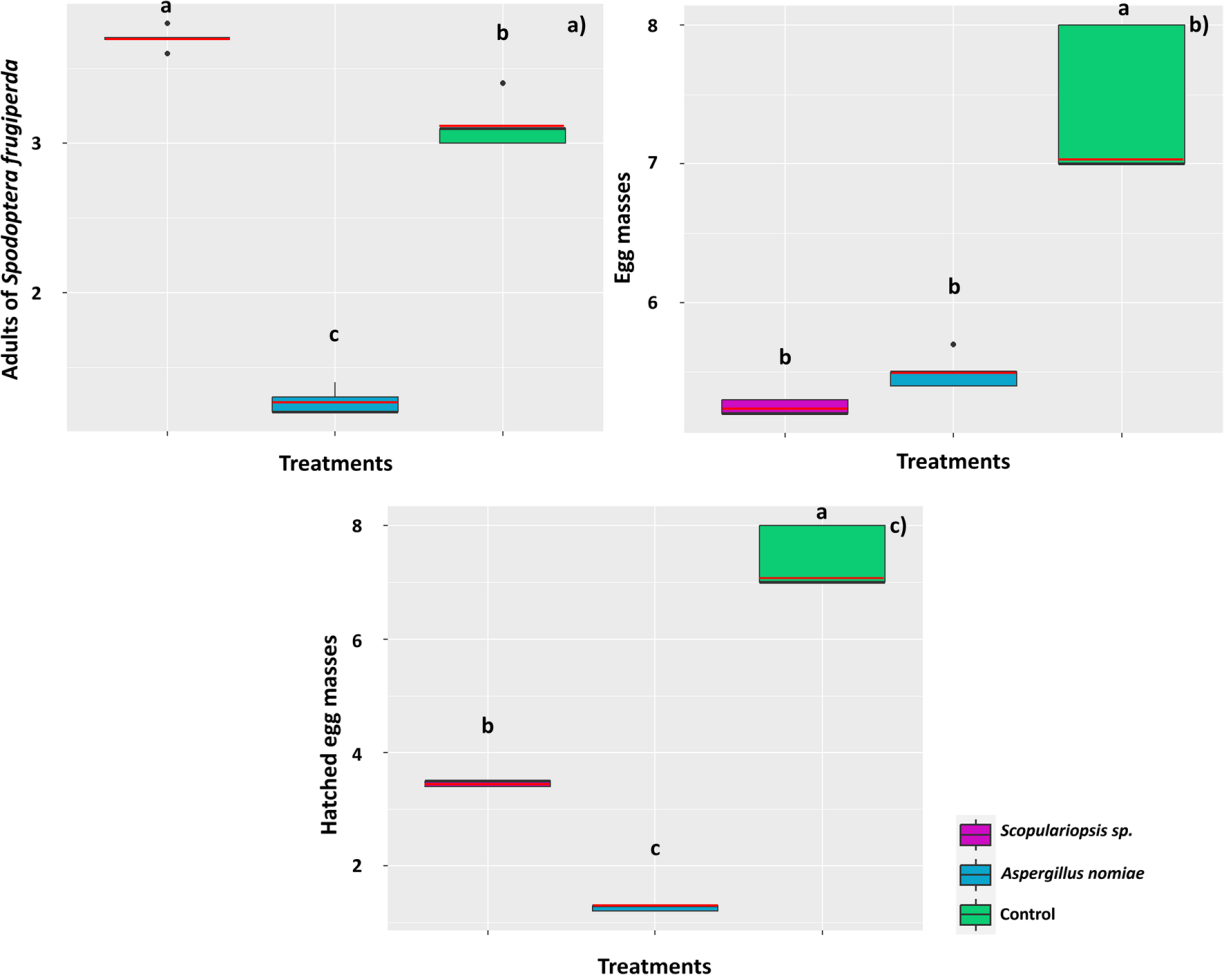
Mortality of *Spodoptera frugiperda* subjected to a diet containing spores of the fungi *Scopulariopsis* sp. and *Aspergillus nomiae* at different stages of development: moths (**a**), egg mass (**b**), and hatched egg mass (**c**). The black horizontal bars within the boxplots represent the median number of dead insects obtained from five replicates with ten initial insects, and the red horizontal bars represent the average. Points outside the box are outliers, and above the boxes, equal letters represent statistically similar means (Tukey’s test, *p* < 0.05). In (b), the images show egg masses contaminated with the two fungi tested and from the control treatment

Regarding adult mortality, insects subjected to a diet containing spores of *Scopulariopsis* sp. had their mortality anticipated (mean = 3.7 ± 0.07 dead moths, equivalent to 37% mortality) compared to the other treatments (*F* = 609.35; D.F. = 14; *p* = 0.000) (Fig. 8a). The diet containing spores directly affected the hatching of the surviving moths (*F* = 65.59; D.F. = 14; *p* = 0.000); they presented a mean of 5.24 ± 0.07 and 5.50 ± 0.12 offsprings, respectively, for those treated with *Scopulariopsis* sp. and *A. nomiae*, whereas the control treatment reached a mean of 7.40 ± 0.54 offsprings (Fig. 8b). Only a mean of 1.26 ± 0.05 offsprings from moths treated with *A. nomiae* hatched (*F* = 474.37; D.F. = 14; *p* = 0.000) (22.9% of total offsprings), and 3.46 ± 0.05 from those treated with *Scopulariopsis* sp. (66% of total offsprings), while all offsprings of the control treatment hatched (Fig. 8c).

**Fig. 9:**
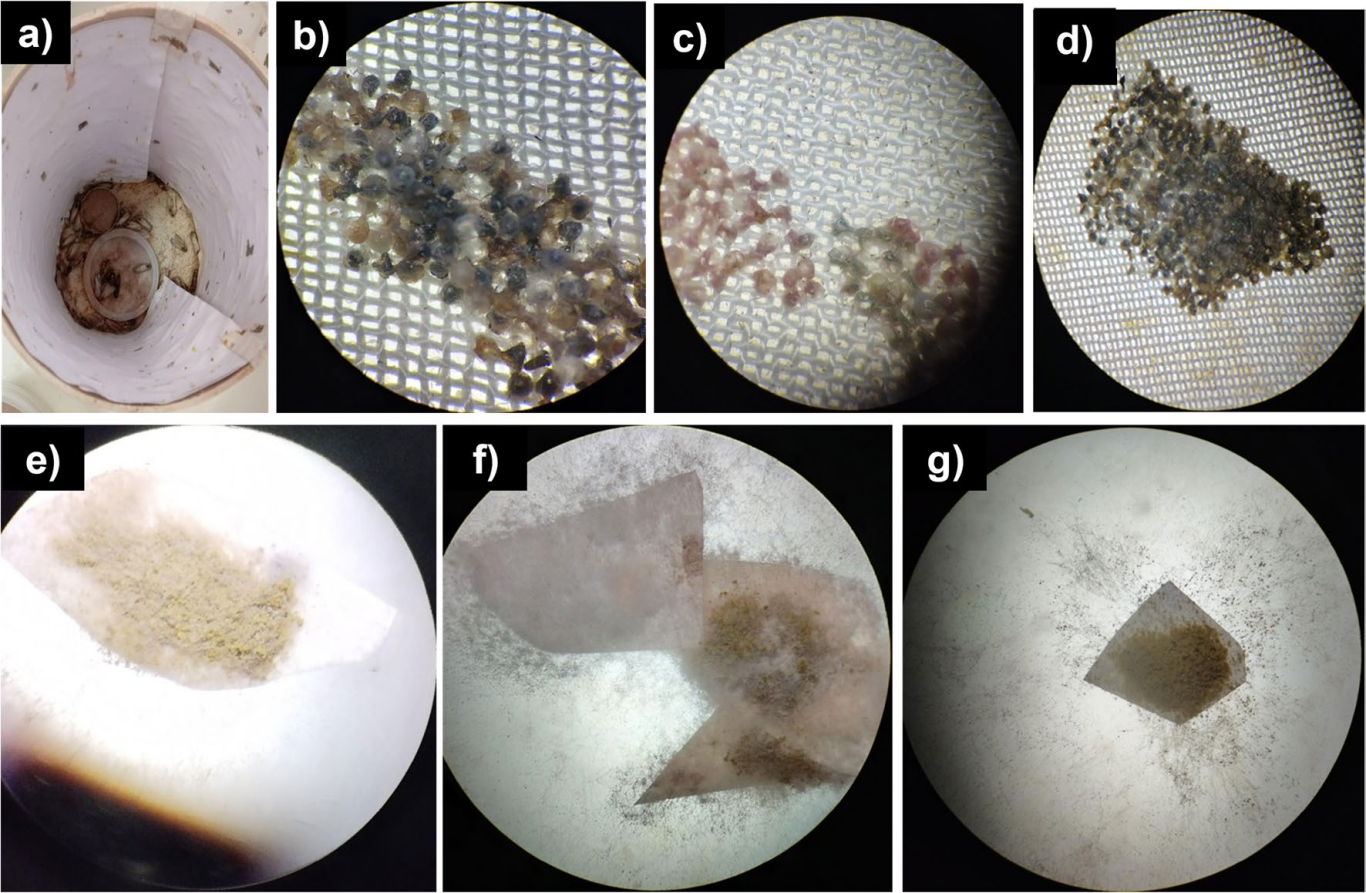
Vertical transmission of spores of the fungi *Scopulariopsis* sp. and *Aspergillus nomiae* to *Spodoptera frugiperda* offsprings subjected to a diet containing spores of these fungi. Cage of moths contaminated with *Scopulariopsis* sp. (**a**); offsprings with mycelial development of *Scopulariopsis* sp. and affected hatching (**b**–**d**); and offsprings with mycelial development of *Aspergillus nomiae* and affected hatching (**e**–**g**)

Some of the incubated offsprings from fungal spore treatments were contaminated, with mycelial growth observed on the eggs (Figs. 8b and 9), confirming the vertical transmission of spores from the moth to the offsprings. Thus, mycelial growth directly affected egg hatching in treatments with *Scopulariopsis* sp. (Fig. 9a–d) and *A. nomiae* (Fig. 9e–g), making them non-viable, especially in the treatment with *A. nomiae*.

### Total Mortality of *A. grandis* Adults Over the Entire Exposure Period

**Fig. 10:**
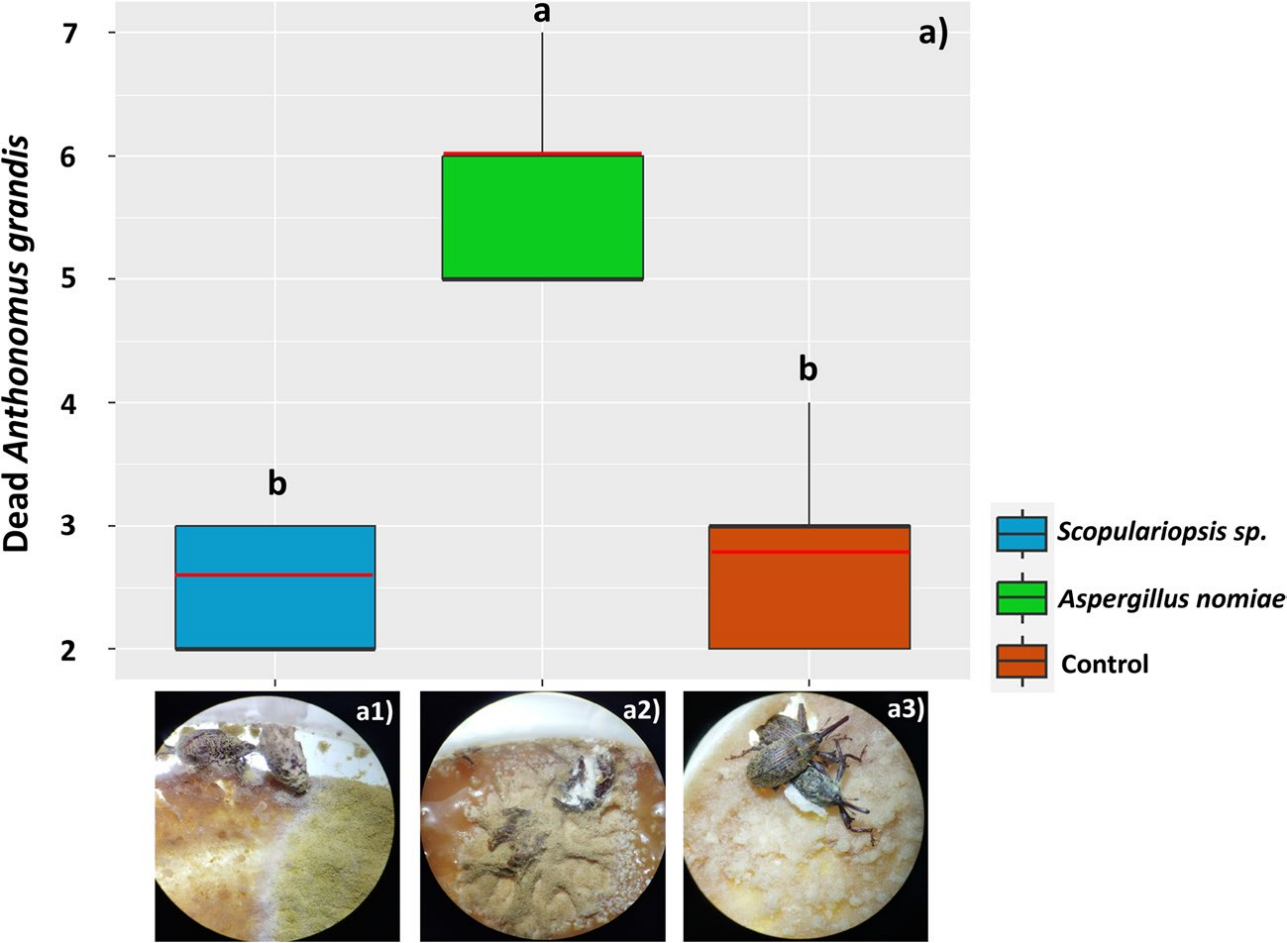
Mortality of *Anthonomus grandis* adults subjected to a diet containing spores of the fungi *Scopulariopsis* sp. and *Aspergillus nomiae* **a**. The black horizontal bars within the boxplots represent the median number of dead insects obtained from 13 replicates with ten initial insects, and the red horizontal bars represent the average. The vertical bars are the maximum and minimum values and above the boxes, equal letters represent statistically similar means (Tukey’s test, *p* < 0.05). The photos show dead insects, colonized respectively by *Scopulariopsis* sp. (a1) and *Aspergillus nomiae* (a2), as well as healthy insects (a3), verified in the control treatment

The treatment with *A. nomiae* in the diet of *A. grandis* affected the survival of adults throughout the exposure period of 30 DAT (*F* = 234.37; D.F. = 38; *p* = 0.000), with a mean of 6 ± 0.70 insects dead by this treatment (representing 46.15% of the test insects). However, *A. grandis* adults were not affected by *Scopulariopsis* sp. spores, as verified over the initial 15 DAT (Fig. 10).

## Discussion

Enterobacter hormaechei has an entomopathogenic effect on S. frugiperda larvae and pupae.

*Spodoptera frugiperda* was affected by the presence of *E. hormaechei* in the diet. The species *E. hormaechei* belongs to the family Enterobacteriaceae, which consists of 23 strains, of which 22 have been isolated from humans. Several epidemiological studies have reported that *E. hormaechei* is the most prevalent species in clinical settings (Ji et al. 2021; Yang et al. 2018; Chavda et al. 2016; Moradigaravand et al. 2016; Morand et al. 2009), being responsible for several infections (Paauw et al. 2009; Mezzatesta and Gona 2012).

*Enterobacter hormaechei* is rarely associated with symptoms in animals. However, some researchers recently associated this bacterium with pathogenesis in foxes, piglets, and calves (Wang et al. 2020a, b; Shan-Shan et al. 2017; Lu-Yao et al. 2017). *Enterobacter hormaechei* is commonly found in many environmental niches, such as sediment and stream water impacted by agricultural activity (Halda-Alija et al. 2001), with concerns that different communities of animals can be hosts with immunological incompetence and are therefore susceptible to *E. hormaechei* infections. Studies have shown that *E. hormaechei* is an important component present in the gastrointestinal tract of insects (Asimakis et al. 2019), and that one of the functions of Enterobacteriaceae in insects is to facilitate the transmission of pathogens (Cirimotich et al. 2011). Thus, since we isolated this bacterium from *S. frugiperda* cadavers and proved its pathogenesis to caterpillars and pupae of this species, *E. hormaechei* may also affect artificial populations of this species through direct transmission from humans handling the insects; or natural populations, through the dispersion of this pathogen in natural environments. Studies on the pathogenicity of *E. hormaechei* in insects are rare, but this species was also isolated from nematode-infected *S. litura* larvae (Manjula et al. 2020), suggesting that the activity of this symbiotic bacterium in caterpillars tissues may facilitate nematode attack, decreasing immune tolerance. Reinhardt et al. (2005) isolated *E. hormaechei* from the bed bug *Cimex lectularius*, and suggested that female insemination by contaminated males is an important source of mortality of bedbugs by entomopathogenic bacteria.

*Enterobacter hormaechei* virulence characteristics involve the ability of strains to invade intestinal epithelial cells and blood–brain barrier endothelial cells and to persist in macrophages (Townsend et al. 2008). These bacteria use several strategies to destroy or actively manipulate the humoral and cellular immune defense mechanisms of insects, which involve the secretion of various enzymes such as proteases, phenoloxidase inhibitors, and toxins that interfere with phagocytosis (Sicard et al. 2008; Hao et al. 2008). These enzymes and toxins facilitate the penetration of other parasites into the host’s hemocoel, acting against the insect’s defense system (Kaya and Gaugler 1993). This explains the virulence effects in *S. frugiperda*.

Enzymes and toxins may constitute the *E. hormaechei* metabolic network of action. Manjula et al. (2020) demonstrated that secondary metabolites of this bacterium extracted in ethyl acetate produce high larval mortality in *S. litura*. Thus, future studies with different *E. hormaechei* strains may propose strategies for the controlled use of this bacterium or of specific culture metabolites to biocontrol *Spodoptera* under agricultural pest conditions.

### Serratia marcescens, *A. nomiae*, and *Scopulariopsis sp*. Affect the Development of *S. frugiperda*

In general, the inclusion of these microbial agents in the diet affected the development of *S. frugiperda*, suggesting metabolic stress in the initial exposure time. This metabolic stress stems from tolerance attempts, with secretory system stress acting as a regulator (Lissner and Schneider 2018). Significant metabolic changes in insects during infectious processes include anorexia (Ayres and Schneider 2009), depletion of glycogen and triglyceride energy stores (Chambers et al. 2012; Dionne et al. 2006), changed insulin signaling (Dionne et al. 2006), and broad metabolite changes (Louie et al. 2016). Thus, these changes reduce energy availability for growth and development, affecting phase changes throughout the insect life cycle and explaining the effects reported in *S. frugiperda*.

Insect ecdysis is closely associated with immune responses (Nunes et al. 2021). This is a result of the hormone 20-hydroxyecdysone triggering a series of molting-related processes in insects, such as apolysis, epidermal cell division, old cuticle digestion, and new cuticle secretion (Nijhout 1994). However, the developmental profile of responses to 20-hydroxyecdysone shows that this hormone also regulates other processes such as metabolism, stress response, and immunity (Nunes et al. 2021; Toprak 2020; Toprak et al. 2020).

The antioxidant system is a vital metabolic pathway during infectious processes in insects, demanding high energy loads (Ramarao et al. 2012). Reactive oxygen species levels increase in the midgut of sandflies fed the bacterium *S. marcescens* (Chaitanya et al. 2016). Several studies confirm the insecticidal potential of *S. marcescens* (Zhang et al. 2021; Wang et al. 2021), suggesting serralysin as a virulence factor synthesized by *S. marcescens*. This factor suppresses cellular immunity by degrading adhesion molecules and increasing bacterial pathogenesis (Lee et al. 2017; Tambong et al. 2014). However, Lee and Lee (2022) demonstrated that some insects can show high detoxification activity against serralysin in the midgut, while the activity of the lytic enzymes protease and phospholipase and of oxidative stress enzymes increases in the gut and also in the hemolymph. This was demonstrated for *S. litura* caterpillars fed a diet supplemented with *S. marcescens* (Aggarwal et al. 2021). Thus, metabolic stress and the need to respond to *S. marcescens*, *A. nomiae*, and *Scopulariopsis* sp. infection affected the development of *S. frugiperda*, possibly through ecdysis hormone-induced pathways. However, an effective detoxifying system reducing the metabolic stress caused by serralysin may explain the weaker mortality effects in the treated *S. marcescens* caterpillars than in those fed *E. hormaechei*. Even though these microorganisms are not known to be insect pathogens, their direct application can weaken the immune system, making it prone to secondary infection by other pathogens.

### *Aspergillus nomiae* Affects the Survival of *S. frugiperda* larvae and *A. grandis* Adults

*Aspergillus nomiae* is not considered a clinically relevant species, although it is closely related to *A. flavus*, which causes opportunistic infections in humans. This indicates that *A. nomiae* is rarely isolated from human or animal infections (Hatmaker et al. 2022). However, *Aspergillus* species are often associated with insect pathogens (e.g., Becchimanzi and Nicoletti 2022). Pollen-consuming insects appear to be most frequently affected, as *Aspergillus* spp. spores contaminate plant pollen that, when consumed by bees, reaches the gut, which is the main site of bee pathogen infection (Foley et al. 2012). Infection by *Aspergillus* species can affect entire insect colonies, resulting in the death of almost the entire colony (Leska et al. 2021). Pathogenicity towards insects may be related to the ability to produce aflatoxins. *Aspergillus nomiae* produces large amounts of the aflatoxins AFB 1, AFB 2, AFG 1, and AFG 2 (Reis et al. 2022). Aflatoxins are a group of furanocoumarins derived from polyketides. They are the most toxic and carcinogenic compounds among the known mycotoxins (Bennett and Klich 2003). However, alternatively, insecticidal aflatoxins produced by *A. nomiae* can impact humans indirectly (Bhardwaj et al. 2023). Therefore, we recommend that this fungus be used only in the management of pests that attack species of agricultural importance, such as textile or timber plants, which are not intended for human or animal food consumption, as a way of avoiding poisoning incidents.

*Aspergillus nomiae* has been mentioned in biocontrol studies for its ability to produce volatile organic compounds (Holkar et al. 2023). Tu et al. (2022) isolated potential entomopathogenic agents from the leaf beetle *Plagiodera versicolor*, including *A. nomiae*, and suggested that this fungus has great potential for the development of a pest management microbial agent. According to those authors, first-instar larvae sprayed with *A. nomiae* spore suspension started to die 12 h after inoculation. Recently, Zhang et al. (2024) isolated a strain of *A. nomiae* from naturally infected *S. litura* caterpillars, and the strain showed strong pathogenicity for five insect pests belonging to Lepidoptera and Hemiptera, in addition to inhibiting the growth of *Sclerotinia sclerotiorum* in vitro. These authors suggest the use of *A. nomiae* for dual biocontrol: insect pests and phytopathogenic fungi. This explains the entomopathogenic effect of *A. nomiae* on *S. frugiperda* and *A. grandis*. Thus, we also suggest toxicological studies and field tests to directly analyze spores of this fungus, or even metabolites extracted from crops, aiming at its use in agricultural pest biocontrol.

Our results are expressive considering the vertical spore transmission to *S. frugiperda* offsprings. Cases of vertical fungus transmission in insects have been described in the literature (Bright and Bulgheresi 2010), such as the transmission of *Fusarium verticillioides* to the offspring of *Diatraea saccharalis*, which continues the cycle by inoculating the fungus in healthy plants. *Fusarium verticillioides* DNA was present throughout the life cycle of caterpillars fed a diet colonized by this fungus (Franco et al. 2021). Other fungi such as *Metarhizium anisopliae* produce sublethal reproductive effects, interfering with oothecal production, ootheca hatchability, and nymphal production in *Blatella germanica* (Quesada-Moraga et al. 2004). Vertical transmission is an important mechanism to supply insect eggs with beneficial microorganisms (e.g., Kaltenpoth and Flórez (2020); Onchuru et al. (2018)). However, other microorganisms such as *Wolbachia* can be transmitted by this route, and transmission can take place through the external surface of eggs or be transovarial, directly through the inside of the eggs (Kellner 2002). Akutse et al. (2020) showed that the oviposition, hatchability, and longevity of *S. frugiperda* larvae are significantly affected in females infected with *M. anisopliae* and *B. bassiana*, two important agricultural pest biocontrol agents. Thus, the vertical transmission of *A. nomiae* from adult females to *S. frugiperda* offsprings may be part of a future strategy for efficient control of this pest that affects not only larvae or pupae, but also directly affects the eggs.

### *Spodoptera frugiperda* and *A. grandis* Adults are Insensitive to the Action of *Scopulariopsis sp*.

*Scopulariopsis* belongs to the group *Hyphomycetes*, and its teleomorphs are included in the genus *Microascus* (order Microascales). These saprobes are commonly isolated from soil, air, plant debris, paper, and wet indoor environments (e.g., Woudenberg et al. (2017); Samson et al. (2010)). Some species are opportunistic pathogens, causing mainly superficial tissue infections and representing some of the main causes of non-dermatophytic onychomycoses (Sandoval-Denis et al. 2013). Although species of this genus have insecticidal properties poorly reported in the literature, Machowicz-Stefaniak et al. (1993) described biocontrol possibilities using the species *Scopulariopsis brevicaulis*, a synonym of *S. insectivora*. *Scopulariopsis* has already been isolated from insect larvae (Sandoval-Denis et al. 2016) or adults (Yoder et al. 2003), apparently being an entomopathogenic agent. *Scopulariopsis brevicaulis* isolated from aphids demonstrated potential for the biocontrol of these hosts (Abdel Galil et al. 2019). Fungal phytopathogens can also be affected by *Scopulariopsis*. Bosso et al. (2016) showed that *S. brumptii* may show antibiotic tendencies to *Phytophthora cinnamomi* and *Phytophthora cambivora* in vitro, and reduce the mortality of *Castanea sativa* seedlings caused by the genus *Phytophthora* in greenhouse experiments.

*Scopulariopsis* sp. had a delayed toxicity effect on *S. frugiperda*, not affecting the insect in the larval or pupal stage, but triggering high mortality in adults, maybe because *Scopulariopsis* sp. produces lower concentrations of the enzymatic arsenal necessary to attack caterpillars and pupae during the insect-fungus interaction. Thus, *A. nomiae* colonizes and kills pupae and caterpillars more quickly, while *Scopulariopsis* sp. reaches higher colonization levels in the adult stage, being also vertically transferred to the offsprings of surviving moths. Filipello Marchisio et al. (2000) reported a rather low enzymatic capacity for keratinolysis in *S. brevicaulis* compared to the efficiency of other keratinolytic fungi, such as those of the genus *Aspergillus* (e.g., Farag and Hassan 2004; Kim 2003). *Aspergillus* sp. produce proteases (Anitha and Palanivelu 2013) such as chitinases, which affect the insect cuticle (Farag et al. 2016; Brzezinska and Jankiewicz 2012). Moharram et al. (2021) showed that *A. niger* can synthesize almost twice the amount of chitinases produced by *S. brevicaulis*. Thus, *A. grandis* adults were also insensitive to *Scopulariopsis* sp., although this fungus has a great capacity to invade the body of the mite *Psoroptes cuniculi*, causing a high mortality (Perrucci et al. 2008) that may be related to the thin and flexible *P. cuniculi* exoskeleton (Sun et al. 2020; Nalepa 2011), which is therefore more susceptible to enzymatic degradation than the hard and resistant carapace of *A. grandis*. We demonstrate that *E. hormaechei* and *A. nomiae* can biocontrol *S. frugiperda* and *A. grandis* under laboratory conditions. Future studies should focus on elucidating the mechanisms underlying the entomopathogenic effect of the analyzed isolates on *S. frugiperda* and *A. grandis,* as well as on the analysis of metabolites, toxicological aspects, and the development of biological formulations that can be applied in the field.

## Conclusions

*Enterobacter hormaechei* and *A. nomiae* isolated from *S. frugiperda* cadavers were pathogenic for this species, affecting caterpillars and pupal survival. *Aspergillus nomiae* also affected the development of *S. frugiperda* in the early stages of exposure, with evidence of vertical spore transfer to offspring and low hatchability. The fungus *Scopulariopsis* sp. does not affect the survival of *S. frugiperda* caterpillars and pupae; however, due to late action, moths and eggs may be affected. *Aspergillus nomiae* also affected the survival of *A. grandis* adults. Therefore, we suggest that the effect of applying *E. hormaechei* and *A. nomiae* be tested on different insects from different orders at large scale and under controlled conditions to investigate their spectrum of action as entomopathogens.

## Data Availability

All the data relevant to this manuscript are available on request from the corresponding author.
